# Racial Disparities in Incidence and Outcomes Among Patients With COVID-19

**DOI:** 10.1001/jamanetworkopen.2020.21892

**Published:** 2020-09-25

**Authors:** L. Silvia Muñoz-Price, Ann B. Nattinger, Frida Rivera, Ryan Hanson, Cameron G. Gmehlin, Adriana Perez, Siddhartha Singh, Blake W. Buchan, Nathan A. Ledeboer, Liliana E. Pezzin

**Affiliations:** 1 Division of Infectious Diseases, Department of Medicine, Medical College of Wisconsin, Milwaukee; 2 Division of General Medicine, Department of Medicine, Medical College of Wisconsin, Milwaukee; 3 Collaborative for Healthcare Delivery Science, Medical College of Wisconsin, Milwaukee; 4 Froedtert Health, Milwaukee, Wisconsin; 5 School of Medicine, Medical College of Wisconsin, Milwaukee; 6 Department of Pathology, Medical College of Wisconsin, Milwaukee; 7 Institute for Health and Equity, Medical College of Wisconsin, Milwaukee

## Abstract

**Importance:**

Initial public health data show that Black race may be a risk factor for worse outcomes of coronavirus disease 2019 (COVID-19).

**Objective:**

To characterize the association of race with incidence and outcomes of COVID-19, while controlling for age, sex, socioeconomic status, and comorbidities.

**Design, Setting, and Participants:**

This cross-sectional study included 2595 consecutive adults tested for COVID-19 from March 12 to March 31, 2020, at Froedtert Health and Medical College of Wisconsin (Milwaukee), the largest academic system in Wisconsin, with 879 inpatient beds (of which 128 are intensive care unit beds).

**Exposures:**

Race (Black vs White, Native Hawaiian or Pacific Islander, Native American or Alaska Native, Asian, or unknown).

**Main Outcomes and Measures:**

Main outcomes included COVID-19 positivity, hospitalization, intensive care unit admission, mechanical ventilation, and death. Additional independent variables measured and tested included socioeconomic status, sex, and comorbidities. Reverse transcription polymerase chain reaction assay was used to test for severe acute respiratory syndrome coronavirus 2 (SARS-CoV-2).

**Results:**

A total of 2595 patients were included. The mean (SD) age was 53.8 (17.5) years, 978 (37.7%) were men, and 785 (30.2%) were African American patients. Of the 369 patients (14.2%) who tested positive for COVID-19, 170 (46.1%) were men, 148 (40.1%) were aged 60 years or older, and 218 (59.1%) were African American individuals. Positive tests were associated with Black race (odds ratio [OR], 5.37; 95% CI, 3.94-7.29; *P* = .001), male sex (OR, 1.55; 95% CI, 1.21-2.00; *P* = .001), and age 60 years or older (OR, 2.04; 95% CI, 1.53-2.73; *P* = .001). Zip code of residence explained 79% of the overall variance in COVID-19 positivity in the cohort (ρ = 0.79; 95% CI, 0.58-0.91). Adjusting for zip code of residence, Black race (OR, 1.85; 95% CI, 1.00-3.65; *P* = .04) and poverty (OR, 3.84; 95% CI, 1.20-12.30; *P* = .02) were associated with hospitalization. Poverty (OR, 3.58; 95% CI, 1.08-11.80; *P* = .04) but not Black race (OR, 1.52; 95% CI, 0.75-3.07; *P* = .24) was associated with intensive care unit admission. Overall, 20 (17.2%) deaths associated with COVID-19 were reported. Shortness of breath at presentation (OR, 10.67; 95% CI, 1.52-25.54; *P* = .02), higher body mass index (OR per unit of body mass index, 1.19; 95% CI, 1.05-1.35; *P* = .006), and age 60 years or older (OR, 22.79; 95% CI, 3.38-53.81; *P* = .001) were associated with an increased likelihood of death.

**Conclusions and Relevance:**

In this cross-sectional study of adults tested for COVID-19 in a large midwestern academic health system, COVID-19 positivity was associated with Black race. Among patients with COVID-19, both race and poverty were associated with higher risk of hospitalization, but only poverty was associated with higher risk of intensive care unit admission. These findings can be helpful in targeting mitigation strategies for racial disparities in the incidence and outcomes of COVID-19.

## Key Points

**Question:**

Is there an association between race and coronavirus disease 2019 (COVID-19) after controlling for age, sex, socioeconomic status, and comorbidities?

**Findings:**

In this cross-sectional study of 2595 patients, positive COVID-19 tests were associated with Black race, male sex, and age 60 years or older. Black race and poverty were associated with hospitalization, but only poverty was associated with intensive care unit admission.

**Meaning:**

The results of this study indicate that in the first weeks of the COVID-19 pandemic in Milwaukee, Wisconsin, Black race was associated with a positive COVID-19 test and the subsequent need for hospitalization, but only poverty was associated with intensive care unit admission.

## Teaser

This cross-sectional study characterizes the association of race with incidence and outcomes of coronavirus disease 2019 (COVID-19), while controlling for age, sex, socioeconomic status, and comorbidities.

## Introduction

The coronavirus disease 2019 (COVID-19) pandemic, caused by novel severe acute respiratory syndrome coronavirus 2 (SARS-CoV-2), has been characterized by substantial geographic variation in incident cases and hospitalizations due to the illness and attributable mortality.^1,2^ In the United States, some areas of intense geographic localization, especially early in the course of the pandemic, were attributable to travel to areas with established disease or to outbreaks related to index cases that had contact with susceptible groups.^3^ As the pandemic became more established with sustained community-based transmission of the virus, these traditional epidemiological risk factors became less obvious as risk factors. Other risk factors for disease incidence and outcomes have emerged, such as age and comorbid health conditions.^4,5^

African American individuals are disproportionally affected by COVID-19 and possibly have worse outcomes of the disease.^6^ Most information on racial disparities in incidence and outcomes related to COVID-19 arise from focused geographic areas, given that national data sources on the disease typically omit information on race. Milwaukee County (Wisconsin) is an area that has demonstrated racial disparities in COVID-19. By April 6, 2020, 601 of 1304 cumulative confirmed cases (46.1%) in Milwaukee County had occurred among African American residents, who represent 27.2% of the county’s population.^7^ By the same date, 33 of 45 deaths (73.3%) due to COVID-19 had occurred in African American residents. African American individuals may experience poorer health care access and a greater burden of comorbid illness, which seems to be a particularly relevant factor in determining the outcomes of COVID-19.^8,9,10,11,12,13,14,15,16^

Racial disparities could also be due to factors associated with lower socioeconomic status (SES), as African American US residents tend to have lower incomes and educational status than White residents.^6,17,18^ Lay reports suggest that individuals residing in zip codes characterized by lower SES are at higher risk for contracting the virus.^19^ This may be because individuals with lower SES are more likely to continue working and taking public transportation despite local governmental stay-at-home policies, presumably because of fewer opportunities to continue employment while working from home. Supporting this hypothesis, location data on cellular telephone users^20^ has found that after implementation of stay-at-home mandates, individuals residing in lower-income areas have been slower to reduce their movements than individuals residing in higher-income areas. It is unclear whether persons with lower SES have worse outcomes once they contract the virus, specifically whether they are more likely to be hospitalized, to require mechanical ventilation, or to die. A May 2020 publication^16^ found that poverty and race were associated with hospitalizations but not with mortality among patients with COVID-19.

The goal of this study was to describe the patterns and outcomes of COVID-19 by race while controlling for age, sex, SES, and comorbid conditions. To accomplish this goal, we used information from the largest academic health system in Wisconsin, located in Milwaukee County. The use of health care facility data allows for the analysis of data elements (ie, race, zip code of residence, comorbid illness) that are not typically available from government public health sources on COVID-19.

## Methods

### Setting

This study was performed at Froedtert Health and the Medical College of Wisconsin, an academic medical system located in southeast Wisconsin comprising 3 acute care hospitals serving a population of 1.8 million individuals. The health system has a total of 40 outpatient clinics and 879 inpatient beds, of which 128 are intensive care unit (ICU) beds. During fiscal year 2019, there were 45 133 admissions and 1 081 830 ambulatory visits. Froedtert Health uses a centralized laboratory (Wisconsin Diagnostics Laboratory) for all tests, including COVID-19 (see testing section). This study was approved by the Medical College of Wisconsin institutional review board, and a waiver of informed consent was granted because of the minimal risk associated with the study and because the research could not practicably be carried out without the waiver. This study followed Strengthening the Reporting of Observational Studies in Epidemiology (STROBE) reporting guideline for cross-sectional studies.^23^

### Study Design and Variable Definitions

This cross-sectional study included all consecutive, unique patients tested for COVID-19 at the study health system from March 12 to March 31, 2020. Patients were identified from the daily enterprise analytic reports. Both inpatients and outpatients were included in the study. Persons identified as health system employees or those younger than 18 years were excluded. Data were collected from the electronic medical records, including demographic characteristics (eg, age, sex, race), presenting symptoms, comorbidities, primary and secondary health insurance, and dates of hospital admission, ICU admission, receipt of mechanical ventilation, and disposition within 14 days of admission (classified as deceased, discharged home, discharged to other institutional setting, or not discharged). Patients admitted to the hospital were followed up until discharge or death. Patients never admitted were followed up for as long as 14 days.

Race was based on self-reported data available in the electronic medical record. Individuals were classified as having African American or other race, which included White, Native Hawaiian or Pacific Islander, Native American or Alaska Native, and Asian. In the absence of information on income, we used lack of health insurance or enrollment in Medicaid, the public program that provides health insurance to individuals with low income or disability, as our individual-level indicator of poverty. Presenting symptoms, collected from medical notes, included presence of fever; cough; shortness of breath; sore throat; diarrhea, nausea, vomiting, or abdominal pain; changes in mental status; and olfactory or taste changes. Comorbidities were collected from the problem list or medical notes; variables abstracted included hypertension, diabetes, chronic heart disease, chronic lung disease, and chronic kidney disease. Medical notes were also the source of information on body mass index (BMI; calculated as weight in kilograms divided by height in meters squared) and history of smoking. Finally, patients’ addresses were used to obtain the 9-digit zip code of residence, with which we classified persons as living in a socially disadvantaged area based on a score of 7 or higher in the Area Deprivation Index abstracted from the Neighborhood Atlas.^21,22^ This threshold was selected because it most closely corresponded to the lowest quintile in state-specific US Census–tract indicators of low SES, such as proportion of persons with incomes at or below the federal poverty level and per capita income. Recognizing the importance of identifying potential hot spots for the transmission of the disease, we leveraged information on patients’ residence in a socially disadvantaged area to provide a visual depiction of COVID-19 positivity as it was associated with a small-area measure of low SES (Figure).

**Figure.**
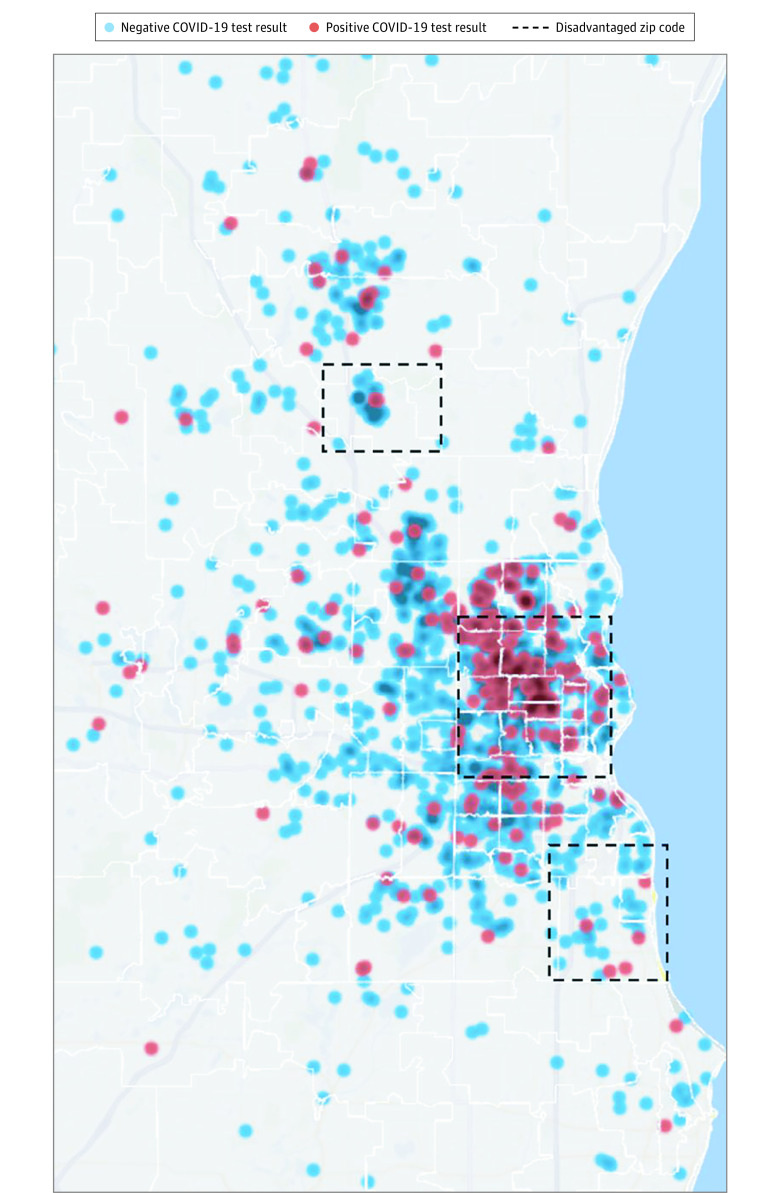
Distribution of Coronavirus Disease 2019 (COVID-19) Cases Based on Socioeconomically Disadvantaged Zip Codes in Milwaukee, Wisconsin Patient postal codes were mapped using blur and jitter to maintain confidentiality and were categorized based on COVID-19 test results. Socially disadvantaged areas are defined as those 9-digit zip codes with a score of 7 or higher in the Area Deprivation Index.^21^ Map created using Excel 2016 3D Maps (Microsoft Corp).

### Test Methods

Starting March 12, COVID-19 tests were performed at the Wisconsin Diagnostics Laboratory using the US Centers for Disease Prevention and Control (CDC) methodology for reverse transcription polymerase chain reaction.^24^ Two swabs were collected from each patient (nasopharyngeal and oropharyngeal specimens) using a minitip and regular-sized flocked swab (Copan Diagnostics), respectively, and placed in viral transport media (M6, ThermoFisher). Testing for SARS-CoV-2 was performed using the emergency use authorization–approved CDC SARS-CoV-2 assay.^24^ RNA was extracted using the eMag (bioMerieux) according to the manufacturers’ product insert. Real-time reverse transcription polymerase chain reaction was performed on ABI 7500 Fast DX thermocyclers (ThermoFisher) according to CDC protocol.^24^

### Testing Algorithm

#### March 12 to 16

Outpatient tests were performed based on the presence of fever and cough and either a history of exposure to a patient with confirmed COVID-19 or history of travel to 1 of the initial hotspots for COVID-19, per CDC recommendations. A virtual triage center (through a centralized phone number) evaluated patients and approved, ordered, and arranged orders based on the previously described testing algorithms. All symptomatic patients who required hospitalization and were aged 65 years or older or had chronic conditions (ie, diabetes, hypertension) were tested. Race was not included as a criterion for testing.

#### March 17 to 31

Outpatient testing was performed for patients presenting with cough or shortness of breath and age older than 60 years or presence of diabetes, chronic lung disease, congestive heart failure, or immunocompromised status (ie, receiving immune modulators, undergoing active cancer treatment, transplantation status, third trimester of pregnancy). Testing in the inpatient setting or in the emergency department was performed for any patient presenting with either cough or shortness of breath of unclear etiology or probable infectious diseases etiology.

### Statistical Analysis

Multivariable, generalized estimating equations (GEE) logit models adjusted for clustering of patients at the 9-digit zip code were used to examine factors associated with a positive test (among all tested), with outcomes of hospital admission, ICU admission, and mechanical ventilation (among patients with COVID-19), and with in-hospital mortality (among hospitalized patients with COVID-19). In addition to offering a sound statistical approach for handling the collinearity resulting from the high correlation of patients’ residence in a socially disadvantaged area with race (r = 0.59) and, to a lesser extent, with our individual-level measure of poverty (Medicaid enrollment and/or lack of insurance; r = 0.33), the GEE technique allowed us to adjust for other, unobserved small-area factors that are shared by patients residing in the same zip code. The choice of GEE covariance structure was based on the method described by Pan.^25^

In addition to race and poverty status, all multivariable models included indicators for age group (≥60 years vs <60 years), sex, comorbidities (ie, 0, 1-2, or ≥3, based on coexisting risk conditions including diabetes, hypertension, heart disease, lung disease, kidney disease, and cancer), presenting symptoms, smoking history, and BMI (including an indicator for missing BMI). Finally, we included an interaction term between race and poverty to test our a priori hypothesis that SES acts as a modifier of the race variable in COVID-19 positivity and disease severity.

Statistical significance was set at α = .05 for 2-tailed tests. All analyses were conducted in Stata version 16 (StataCorp).

## Results

A total of 2595 patients were included in the analysis (eFigure 1 in the Supplement); 785 (30.2%) were African American individuals, 1617 (62.3%) were White individuals, and 193 (7.4%) belonged to other racial groups. Table 1 presents summary statistics for the cohort, overall and by race. The mean (SD) age of the combined group was 53.8 (17.5) years, 978 (37.7%) were men, and the mean (SD) BMI was 31.5 (8.2), with 1349 (52.0%) meeting the definition of obesity. Overall, 716 (27.6%) met the definition of poverty (ie, uninsured or Medicaid beneficiary). Nearly 80% had at least 1 comorbid condition associated with higher COVID-19 risk (2052 [79.1%]); 631 (24.3%) had 3 or more such risk factors.

**Table 1.** Characteristics of Persons Tested for COVID-19, Overall and by Race

Characteristic	Patients, No. (%)			*P* value
	Total (N = 2595)	Other racial group (n = 1810)^a^	African American (n = 785)	
Age, y				
Mean (SD)	53.8 (17.5)	54.9 (17.7)	51.3 (16.7)	.001
≥60	1004 (38.7)	763 (42.2)	241 (30.7)	.001
Men	978 (37.7)	708 (39.1)	270 (34.4)	.02
Poverty status, ie, uninsured or receiving Medicaid	716 (27.6)	328 (18.1)	388 (49.4)	.001
Comorbidities^b^				.001
0	543 (20.9)	394 (21.8)	149 (19.0)	
1-2	1421 (54.7)	1011 (55.9)	410 (52.2)	
≥3	631 (24.3)	405 (22.4)	226 (28.9)	
BMI, mean (SD)	31.5 (8.2)	30.8 (7.8)	33.2 (8.8)	.001
Missing BMI	371 (14.3)	125 (15.9)	246 (13.6)	.13
History of smoking or current smoker	1151 (44.3)	821 (45.4)	330 (42.0)	.11
Symptoms				
Fever	667 (25.7)	451 (24.9)	216 (27.5)	.16
Cough	1690 (65.1)	1168 (64.5)	522 (66.5)	.33
Shortness of breath	1157 (44.6)	768 (42.4)	389 (49.6)	.001
Diarrhea, nausea, vomiting, or abdominal pain	321 (12.4)	177 (9.8)	144 (18.3)	.001
Other symptoms	514 (19.8)	357 (19.7)	157 (20.0)	.86
Outcomes				
COVID-19 positive	369 (14.2)	151 (8.3)	218 (27.8)	.001

Abbreviations: BMI, body mass index (calculated as weight in kilograms divided by height in meters squared); COVID-19, coronavirus disease 2019.

^a^ Other racial group included White, Native Hawaiian or Pacific Islander, Native American or Alaska Native, and Asian patients as well as patients with unknown racial identification.

^b^ Comorbidities were categorized based on presence of diabetes, hypertension, heart disease, lung disease, kidney disease, and cancer, excluding skin malignancies.

Of the 116 patients with COVID-19 who were admitted, 26 (22.4%) were tested in an outpatient clinic, and 87 (75.0%) were tested on the same day of hospital admission in the emergency department. Only 3 patients (2.6%) were already admitted to the hospital when a COVID-19 test was ordered. Of the 253 patients with COVID-19 who did not require hospitalization, 155 (61.3%) were tested in an outpatient clinic, and 98 (38.7%) were tested in the emergency department.

The African American group was younger than the group of patients who belonged to other racial groups, with nearly 70% being younger than 59 years (543 [69.2%] vs 1047 [57.8%]; *P* = .001). Overall, 388 African American patients (49.4%) were poor, compared with 328 patients (18.1%) who belonged to other races (*P* = .001). The proportion of African American patients with 3 or more comorbidities was higher than that of patients who belonged to other racial groups (226 [28.9%] vs 405 [22.4%]; *P* = .001) as was mean (SD) BMI (33.2 [8.8] vs 30.8 [7.8]; *P* = .001). Among African American patients with known BMI, 492 (62.6%) were obese compared with 862 (47.6%) among patients from other racial groups. With the exception of a higher prevalence of shortness of breath (389 [49.6%] vs 768 [42.4%]; *P* = .001) and gastrointestinal symptoms (144 [18.3%] vs 177 [9.8%]; *P* = .001) among African American patients compared with all other patients, there were no differences in symptoms between the 2 groups. African American patients were approximately 3 times more likely to test positive for COVID-19 than persons from other racial groups (218 [27.8%] vs 151 [8.3%]) (eFigure 2 in the Supplement).

### Race, SES, and COVID-19 Positivity

Multivariable estimates shown in Table 2 indicate that, regardless of SES, African American patients were considerably more likely to test positive for the virus than persons of other races (odds ratio [OR], 5.37; 95% CI, 3.94-7.29; *P* = .001), even after controlling for differences in demographic and health characteristics, comorbidities, presenting symptoms, and clustering at the zip-code level. Male sex (OR, 1.55; 95% CI, 1.21-2.00; *P* = .001) and age 60 years or older (OR, 2.04; 95% CI, 1.53-2.73; *P* = .001) were also associated with COVID-19 positivity. Poverty status was not significant (OR = 0.66; CI = 0.41-1.08; *P* = .10). There was no significant interaction term between race and the poverty indicator, suggesting that SES did not affect differentially the likelihood of a positive test across racial groups. The estimate of the intracluster correlation indicates that zip code of residence explained 79% of the overall variance in COVID-19 positivity in the cohort (ρ = 0.79; 95% CI, 0.58-0.91; *P* = .001).

**Table 2.** Factors Associated With Coronavirus Disease 2019 Positivity

Factor	OR (95% CI)^a^	*P* value
Patient race and socioeconomic status		
Black race	5.37 (3.94-7.29)	.001
Enrolled in Medicaid	0.66 (0.41-1.08)	.10
Interaction of race × poverty status	0.90 (0.49-1.62)	.72
Demographic characteristics		
Age, y		
<60	1 [Reference]	NA
≥60	2.04 (1.53-2.73)	.001
Sex		
Women	1 [Reference]	NA
Men	1.55 (1.21-2.00)	.001

Abbreviations: NA, not applicable; OR, odds ratio.

^a^ The ORs and 95% CIs were obtained from a model estimated using a generalized estimating equation logit specification that adjusts for clustering at the 9-digit zip code–level of patients’ residence. All models also include a constant term and indicators for comorbidities (categorized as 0, 1-2, ≥3, based on presence of diabetes, hypertension, heart disease, lung disease, kidney disease, and cancer); symptoms at testing (categorized as fever, cough, shortness of breath, and other symptoms, including gastrointestinal symptoms); body mass index, including an indicator for missing body mass index; and history of smoking. The intracluster correlation coefficient, estimated based on an exchangeable-correlation covariance structure, had a ρ of 0.79 (95% CI, 0.58-0.91).^24^

### Characteristics of Patients with COVID-19

African American patients testing positive for COVID-19, compared with patients who belonged to other racial groups, were more likely to be younger (≥60 years, 78 of 218 [35.8%] vs 70 of 151 [46.4%]; *P* = .04), to have no insurance or receive Medicaid (81 [37.1%] vs 18 [11.9%]; *P* = .001), to have 3 or more comorbidities (60 [27.5%] vs 25 [16.5%]; *P* = .03), to have higher mean (SD) BMI (33.2 [8.8] vs 30.8 [7.8]; *P* = .007), and to present with shortness of breath (125 [57.3%] vs 69 [45.7%]; *P* = .03) and gastrointestinal symptoms (62 [28.4%] vs 24 [15.8%]; *P* = .005) at testing (Table 3). Among individuals with COVID-19, 116 (31.4%) were hospitalized, 70 (19.0%) required ICU admission, 26 (7.0%) required mechanical ventilation, and 20 (17.2%) died in hospital within 14 days of admission.

**Table 3.** Characteristics of Patients with Coronavirus Disease 2019, Overall and by Race

Characteristic	Patients, No. (%)			*P* value
	Total (N = 369)	Other racial group (n = 151)^a^	African American (n = 218)	
Age, y				
Mean (SD)	54.5 (16.2)	55.6 (16.3)	53.8 (16.1)	.29
≥60	148 (40.1)	70 (46.4)	78 (35.8)	.04
Men	170 (46.1)	77 (51.0)	93 (42.7)	.12
Poverty status, ie, uninsured or receiving Medicaid	99 (26.8)	18 (11.9)	81 (37.1)	.001
Comorbidities				
0	98 (26.5)	47 (31.1)	51 (23.4)	.03
1-2	186 (50.4)	79 (52.3)	107 (49.1)	
≥3	85 (23.0)	25 (16.5)	60 (27.5)	
BMI, mean (SD)	31.5 (8.1)	30.8 (7.8)	33.2 (8.8)	.007
Missing BMI	58 (15.7)	39 (17.9)	19 (12.6)	.16
History of smoking or current smoking	124 (33.6)	57 (37.7)	67 (30.7)	.16
Symptoms				
Fever	187 (50.7)	75 (49.7)	112 (51.4)	.75
Cough	293 (79.4)	120 (79.4)	173 (79.4)	>.99
Shortness of breath	194 (52.6)	69 (45.7)	125 (57.3)	.03
Diarrhea, nausea, vomiting, or abdominal pain	86 (23.3)	24 (15.8)	62 (28.4)	.005
Other symptoms	77 (20.9)	30 (19.9)	47 (21.6)	.69
Outcomes				
Inpatient admission	116 (31.4)	38 (25.2)	78 (35.8)	.03
ICU admission	70 (19.0)	26 (17.2)	44 (20.2)	.47
Mechanical ventilator	26 (7.0)	11 (7.3)	15 (6.9)	.88
In-hospital death^b^	20 (17.2)	6 (15.9)	14 (17.9)	.62

Abbreviations: BMI, body mass index (calculated as weight in kilograms divided by height in meters squared); ICU, intensive care unit.

^a^ Other racial group included White, Native Hawaiian or Pacific Islander, Native American or Alaska Native, and Asian patients as well as patients with unknown racial identification.

^b^ In-hospital death was calculated for the subset of 116 patients with COVID-19 who were hospitalized.

### Race, SES, and COVID-19 Severity of Disease

Both Black race (OR, 1.85; 95% CI, 1.00-3.67; *P* = .04) and poverty (OR, 3.84; 95% CI, 1.20-12.30; *P* = .02) were significantly associated with increased likelihood of hospitalization among the 369 individuals with COVID-19 (Table 4). Among those admitted, poverty, but not race, was significantly associated with ICU admission (Table 4). Poverty increased by more than 3-fold the odds of ICU admission (OR, 3.58; 95% CI, 1.08-11.80, *P* = .04). Except for differential hospitalization rates (78 [35.8%] vs 38 [25.2%]; *P* = .03), there were no statistically significant differences between African American patients and patients from other racial groups in ICU admission (44 [20.2%] vs 26 [17.2%]; *P* = .47), mechanical ventilation (15 [6.9%] vs 11 [7.3%]; *P* = .88), or in-hospital mortality among those admitted (14 [17.9%] vs 6 [15.9%]; *P* = .62) (Table 3). Although the race coefficient was not statistically significant at conventional levels, the coefficient for the interaction term between race and SES (OR, 0.15; 95% CI, 0.03-0.70; *P* = .02), which captures the difference-in-difference of the associations of poverty between the 2 race groups, showed that among patients who belong to other racial groups, the odds of ICU admission were nearly 4 times higher if the person was poor; among African American persons, poverty increased these odds by 50%.

**Table 4.** Hospitalization, ICU Admission, Use of Mechanical Ventilator, and Death Among Patients With Coronavirus Disease 2019^a^

Factor	Hospitalization		ICU admission		Mechanical ventilator		In-hospital death	
	OR (95% CI)^b^	*P* value	OR (95% CI)^b^	*P* value	OR (95% CI)^b^	*P* value	OR (95% CI)^b^	*P* value
Patient race and socioeconomic status								
Black race	1.85 (1.0-3.67)	.04	1.52 (0.75-3.07)	.24	1.02 (0.37-2.80)	.96	1.43 (0.14-14.1)	.31
Enrolled in Medicaid	3.84 (1.20-12.3)	.02	3.58 (1.08-11.8)	.04	2.05 (0.28-14.68)	.47	5.33 (014-19.92)	.36
Interaction of race × poverty status	0.35 (0.09-1.43)	.09	0.15 (0.03-0.70)	.02	0.13 (0.01-1.47)	.10	0.02 (0.001-2.44)	.11
Patient demographic characteristics								
Aged ≥60 y v <60 y	1.78 (1.00-3.18)	.05	1.95 (1.01-3.83)	.05	1.74 (0.67-4.59)	.26	22.79 (3.38-53.81)	.001
Men vs women	1.22 (0.70-2.10)	.48	1.03 (0.59-1.83)	.90	0.44 (0.16-1.15)	.10	0.05 (0.005-0.43)	.06
Comorbidities								
3	1 [Reference]	NA	1 [Reference]	NA	1 [Reference]	NA	1 [Reference]	NA
0	0.43 (0.19-0.98)	.04	0.36 (0.14-0.91)	.03	0.22 (0.05-0.830	.02	0.05 (0.005-0.39)	.005
1-2	0.51 (0.28-0.97)	.04	0.57 (0.29-1.12)	.10	0.25 (0.08-0.70)	.009	0.01 (0.006-0.16)	.001
BMI	0.99 (0.71-2.10)	.55	1.03 (0.99-1.08)	.90	1.06 (1.02-1.09)	.003	1.19 (1.05-1.35)	.006
History of smoking or current smoking vs no smoking	1.38 (0.79-2.43)	.37	2.14 (1.15-3.98)	.01	4.24 (1.54-8.61)	.001	7.15 (0.55-9.22)	.13
Symptoms at testing								
Fever	2.56 (1.49-4.38)	.001	1.87 (1.08-3.46)	.05	2.68 (0.99-7.37)	.06	0.73 (0.15-3.48)	.70
Cough	0.44 (0.24-0.81)	.008	0.65 (0.31-1.34)	.24	0.24 (0.07-0.79)	.02	0.04 (0.005-0.33)	.003
Shortness of breath	3.44 (2.03-5.84)	.001	4.50 (2.31-8.74)	.001	9.86 (2.82-34.39)	.001	10.67 (1.52-25.54)	.02
Diarrhea, nausea, vomiting, or abdominal pain	1.25 (0.69-2.29)	.28	1.16 (0.60-2.25)	.65	1.00 (0.31-3.17)	.99	0.43 (0.09-2.04)	.21
Other symptoms	1.41 (0.75-2.64)	.45	1.36 (0.68-2.25)	.38	3.10 (0.53-18.02)	.21	3.15 (1.10-8.94)	.03

Abbreviations: BMI, body mass index; ICU, intensive care unit; OR, odds ratio.

^a^ Sample size was 369 patients with coronavirus disease 2019 for hospitalization and ICU use; 311 patients with coronavirus disease 2019 for mechanical ventilator, after excluding persons with missing BMI due to collinearity; and 116 hospitalized patients with coronavirus disease 2019 for in-hospital mortality.

^b^ The ORs and 95% CIs were obtained from models estimated using a generalized estimating equation logit specification that adjusts for clustering at the zip code–level of patients’ residence. All models also include a constant term and an indicator for missing BMI.

Results for mechanical ventilation and death indicated that neither race nor poverty were significantly associated with these outcomes. Smoking history (OR, 4.24; 95% CI, 1.54-8.61; *P* = .001) and higher BMI (OR, 1.06; 95% CI, 1.02-1.09; *P* = .003) were associated with mechanical ventilation. Older age (≥60 years: OR, 22.79; 95% CI, 3.38-53.81; *P* = .001), having 3 or more comorbidities (eg, 1-2 comorbidities: OR, 0.01; 95% CI, 0.006-0.16; *P* = .001), and having shortness of breath (OR, 10.67; 95% CI, 1.52-25.54; *P* = .02) or less prevalent, atypical symptoms (eg, changes in mental status, olfactory or taste changes) (OR, 3.15; 95% CI, 1.10-8.94; *P* = .03) at presentation significantly increased the likelihood of death. Higher BMI was also significantly associated with death, with each BMI unit increasing the odds of mortality by 19% (OR, 1.19; 95% CI, 1.05-1.35; *P* = .006).

## Discussion

In this cross-sectional study of a large health system in Milwaukee, Wisconsin, both the rate of testing for COVID-19 infection and the likelihood of a positive result for COVID-19 were substantially higher among African American patients than among persons of other racial groups. Findings from multivariable regressions that adjusted for patients’ clustering within socially disadvantaged zip codes as well as other factors indicate that, although race was strongly associated with COVID-19 positivity and hospital admission, it did not significantly increase the risk of ICU admission, mechanical ventilation, or death. In contrast, poverty was an independent factor associated with higher risk of inpatient hospitalization and ICU admission. Neither race nor poverty contributed to increased risk of mechanical ventilation or death among those with COVID-19 infection.

These findings imply that the adverse outcomes and greater population mortality associated with Black race early in the course of the US pandemic were primarily attributable to greater incidence of COVID-19 among African American residents rather than worse survival once hospitalized, at least when accounting for demographic characteristics and comorbid conditions. The findings of no racial disparities in ICU admission, mechanical ventilation, and death among the group hospitalized with COVID-19 are consistent with other published findings on race and COVID-19 outcomes.^16,26^ For example, a study based in New York City^26^ found no racial disparity associated with the use of invasive mechanical ventilation. Another study based in Louisiana^16^ found that although 77% of the hospitalized patients were African American individuals (compared with 31% of the underlying population), Black race was not associated with higher in-hospital mortality than White race after controlling for sociodemographic and clinical characteristics on admission.

Social distancing is clearly an important factor in determining infection rates with SARS-CoV-2, the causative agent of COVID-19. It is possible that African American individuals are less able to practice social distancing than White individuals. A reason for this could be racial disparity in housing. Data from the US Census Bureau for late 2019 showed that only 44% of African American US residents owned their own home, compared with 74% of non-Hispanic White residents.^27^ In addition, racial bias in mortgage lending has been documented in Milwaukee and other metropolitan areas that exhibit racial disparities in COVID-19 outcomes.^28^ Racial bias in mortgage lending may lead to greater housing density among African American residents, in turn leading to less social distancing. Supporting this contention, persons residing in urban counties rated by the CDC as socially susceptible (in terms of SES, household composition, minority status or language, and housing and transportation) had a greater likelihood of COVID-19 cases and deaths than persons living in less susceptible counties.^29^ African American individuals also make up a large part of the essential workforce. Because essential workers often cannot conduct work at home, social distancing is inherently more difficult for these workers, who also often lack sufficient supplies of personal protective equipment.^30^

In our study, zip code explained 79% of the variance of COVID-19 positivity, indicating that where a patient lived was strongly associated with testing positive. This association was not characterized in a study that evaluated race and poverty in association with COVID-19^16^; however, our data concur with their findings, showing that both Black race and poverty were associated with greater hospitalizations.^16^

The fact that COVID-19 rates were lower among those with Medicaid or no health insurance was surprising, given that most US adults with Medicaid are employed, in many cases as essential workers.^31^ Several factors may have contributed to the higher hospitalization and ICU admission rates among poor persons regardless of race. It is plausible that poor individuals presented for care later in their illness. Poverty may also have influenced these outcomes through factors that we were unable to measure, such as a greater exposure to SARS-CoV-2 viral load, or to underlying physical or immunologic factors. It is worth noting that in the Milwaukee area in March, neither ICU beds nor ventilator supplies were outstripped by demand, presumably owing in part to the relatively early imposition of a state mandate to enforce social distancing measures.^32^ Therefore, neither rationing of ICU care nor ventilator availability was likely a factor in the outcomes described herein. Consistent with other published results,^13,26^ our multivariable analyses indicate that a history of smoking and higher BMI were each independently associated with higher likelihood of use of mechanical ventilation, and higher BMI was independently associated with death.

### Limitations

This study is limited by its relatively short accrual time and dependence on medical records data. Although self-reported, information on race was collected during clinical care, without the specificity that would be typical of a research protocol. That said, the racial distribution of the study cohort is consistent with public health reports for the local area (Figure). The ambulatory testing algorithm favored inclusion of persons with certain comorbidities. While the algorithms may have led to greater testing and COVID-19 positivity among those with these comorbidities, the standardization of the algorithms and the use of these comorbidities as control variables in the analysis should have prevented biased estimates by race of clinical outcomes. Additionally, patients who were not admitted to the hospital were followed up for 14 days; thus, the outcome may have been misclassified because either hospitalizations or deaths may have occurred elsewhere. Furthermore, the study is based on the experience of a single health system, which may limit its generalizability to other geographic areas.

## Conclusions

The results of this study indicate that the increased burden of COVID-19 among African American patients in this large cohort was predominately attributable to a greater incidence of infection. Although African American patients had an increased risk of hospitalization, there were no racial differences in other indicators of disease severity, such as ICU admission, mechanical ventilation, or death. This information is potentially encouraging, given that it suggests no inherent racial susceptibility to poor outcomes from this disease; rather, the burden of this illness among African American individuals may be mitigated by reducing the rate of infection using a combination of established and novel public health methods. Racial disparities associated with COVID-19 should not be used to propagate myths related to racial biology.^33,34^ The recognition of an association between SES and COVID-19 outcomes will likely be fruitful in developing and implementing mitigating measures.

## Notes

Supplement. **eFigure 1. **Flowchart Depicting Excluded Patients **eFigure 2. **Proportion of COVID-19 Results by Racial Group
