# Supplementary material for: Racial Disparities in Incidence and Outcomes Among Patients With COVID-19
Source: JAMA Netw Open. 2020 Sep 25;3(9):e2021892. doi: 10.1001/jamanetworkopen.2020.21892 (PMC7519420; doi:10.1001/jamanetworkopen.2020.21892)
Supplement: Supplement. — eFigure 1. Flowchart Depicting Excluded Patients eFigure 2. Proportion of COVID-19 Results by Racial Group [file jamanetwopen-e2021892-s001.pdf]

Supplementary Online Content

Muñoz-Price LS, Nattinger AB, Rivera F, et al. Racial disparities in incidence and outcomes among patients with COVID-19. *JAMA Netw Open*. 2020;3(9):e2021892. doi:10.1001/jamanetworkopen.2020.21892

eFigure 1. Flowchart Depicting Excluded Patients

eFigure 2. Proportion of COVID-19 Results by Racial Group

This supplementary material has been provided by the authors to give readers additional information about their work.

© 2020 Muñoz-Price LS et al. *JAMA Network Open.*

eFigure 1. Flowchart Depicting Excluded Patients

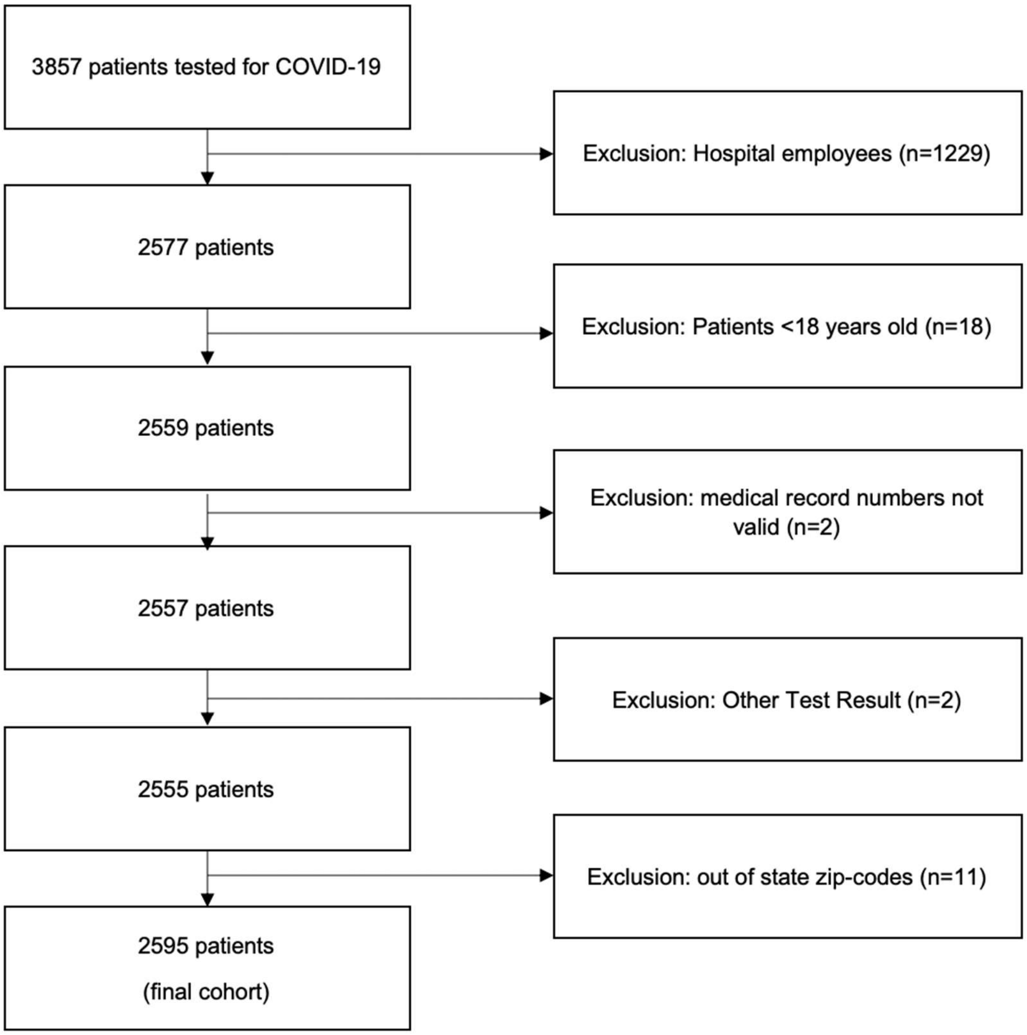

© 2020 Muñoz-Price LS et al. *JAMA Network Open.*

eFigure 2. Proportion of COVID-19 Results by Racial Group

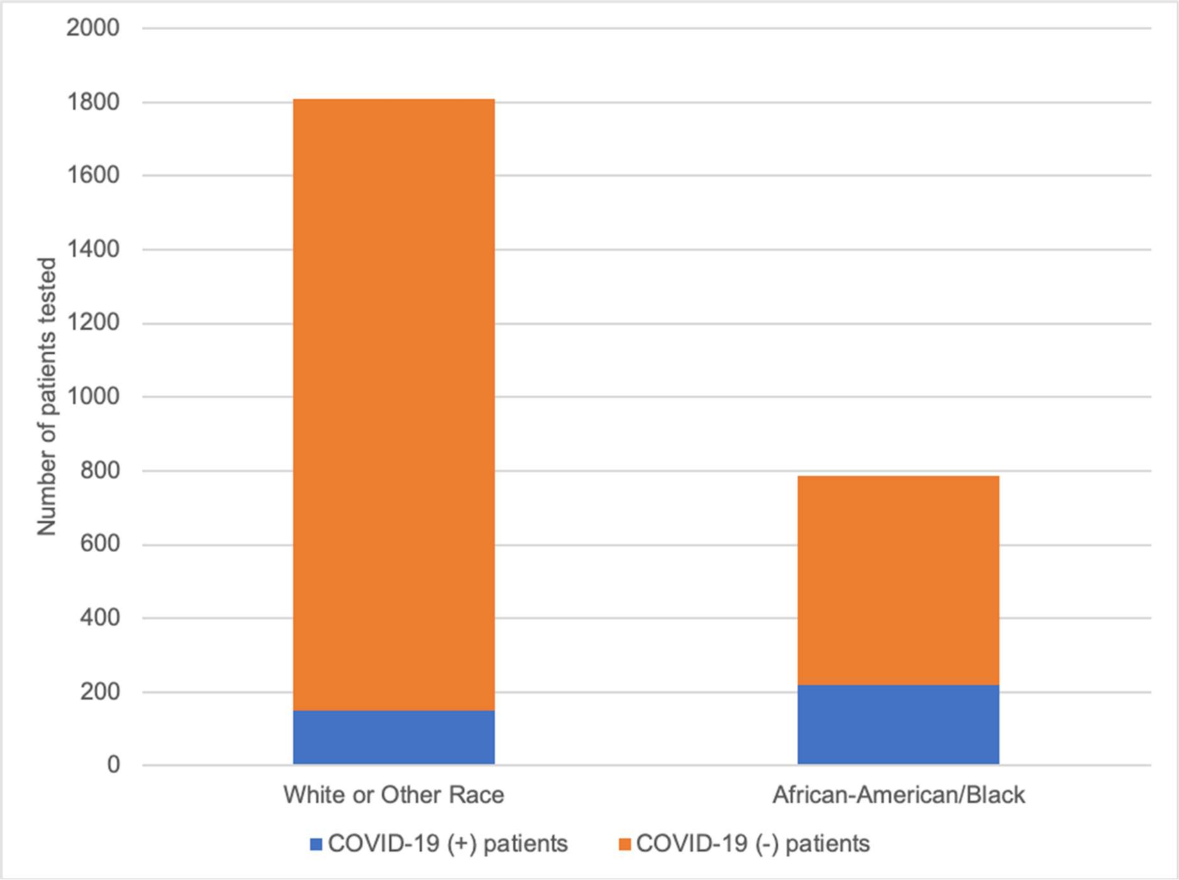

© 2020 Muñoz-Price LS et al. *JAMA Network Open.*
